# The Role of Demographic, Clinical, and Laboratory Characteristics in Predicting the In-Hospital Outcomes of Patients With COVID-19

**DOI:** 10.7759/cureus.23418

**Published:** 2022-03-23

**Authors:** Diyaa H Bokhary, Nidal H Bokhary, Lamees E Seadawi, Ahlam M Moafa, Hashim H Khairallah, Abdullah Bakhsh

**Affiliations:** 1 Department of Emergency Medicine, King Abdulaziz University Hospital, Jeddah, SAU; 2 Department of Family Medicine, King Abdulaziz University Hospital, Jeddah, SAU; 3 Faculty of Medicine, King Abdulaziz University, Jeddah, SAU

**Keywords:** disposition, in-hospital outcome, severity of disease, novel coronavirus, covid 19

## Abstract

Objective

In this study, we aimed to analyze the role of initial patient characteristics obtained at admission (including sociodemographic, clinical, and laboratory findings) in predicting the outcomes in patients with coronavirus disease 2019 (COVID-19).

Methods

This descriptive, retrospective cohort study included all hospital-admitted COVID-19-confirmed cases at a tertiary academic center in Jeddah, the Kingdom of Saudi Arabia (KSA), from March to June 2020. A total of 656 patients with a mean age of 50 ± 19.4 years were included.

Results

Of all the patients recruited, 19.3% required ICU admission, and 19% required mechanical ventilation. The majority (79.9%) of the patients recovered from COVID-19 and were discharged, while 20.1% of them died. Patients with advanced age (p=0.005), male sex (p=0.009), low platelet counts (p=0.015), low hemoglobin levels (p=0.004), low albumin levels (p=0.003), high alkaline phosphatase levels (p=0.002), high blood urea nitrogen levels (p<0.001), and high lactate dehydrogenase levels (p<0.001) were more likely to die.

Conclusion

Based on our findings, it can be inferred that mortality in COVID-19 is highly associated with advanced age and male gender, low platelet counts, low hemoglobin levels, low albumin levels, high alkaline phosphatase levels, high blood urea nitrogen levels, high lactate dehydrogenase levels, tachypnea, and requirement for mechanical ventilation.

## Introduction

Since December 2019, the world has been reeling from the effects of a new kind of coronavirus infection called coronavirus disease 2019 (COVID-19), caused by severe acute respiratory syndrome coronavirus 2 (SARS‐CoV‐2). This disease originated in Wuhan, China, and has spread around the world rapidly and been called a global pandemic [1]. On January 30, 2020, the World Health Organization (WHO) declared the SARS‐CoV‐2 outbreak a public health emergency of international concern. According to the latest statistical data from the WHO, 440,807,756 patients have acquired COVID-19 during this global pandemic so far, resulting in 5,978,096 deaths [2]. 

COVID-19, which has a wide range of symptoms, can affect all age groups. The most common symptoms reported by COVID-19 patients are fever, cough, fatigue, and myalgia [3]. However, some patients may present with non-specific symptoms, such as headache and dizziness [4]. Recent studies have shown that elderly patients with multiple comorbidities and high levels of inflammatory markers usually have a higher mortality risk than others [5,6]. A recent study published in March 2021 showed that male sex, lymphopenia, elevated C-reactive protein (CRP), and presence of comorbidities are all potential risk factors for poor outcomes in COVID-19 patients [7]. In addition, another study found that most COVID-19 patients who presented to the ED with unstable vital signs, most commonly high respiratory rate, had a complicated hospital course that mostly led to ICU admissions and death [8].

To date, multiple studies have confirmed the strong relationship of clinical data and laboratory findings with the general disposition of COVID-19 patients. However, the role of combined clinical and laboratory characteristics in predicting COVID-19 outcomes has not been well described in the Kingdom of Saudi Arabia (KSA). In light of this, the primary objective of this study was to examine how different initial sociodemographic, clinical, and laboratory characteristics in patients could help predict COVID-19 disease outcomes.

## Materials and methods

The institutional ethical board of the King Abdulaziz University Hospital (KAUH) approved this retrospective study and the requirement for informed patient consent was waived. We retrospectively collected and analyzed the data of the 656 patients who were admitted to KAUH with confirmed COVID-19 infection during the period of four months from March to June 2020. We included all patients who presented to the ED with a confirmed COVID-19 diagnosis and required admission to the hospital. COVID-19 infection was diagnosed using real-time polymerase chain reaction (RT-PCR) on a nasopharyngeal sample.

Data were collected using an electronic Google Form. Demographic, clinical, and laboratory data were extracted from the electronic medical records system. Demographic information collected included age, gender, nationality, and comorbidities. With regard to clinical parameters, we documented data on presenting symptoms, such as fever, cough, shortness of breath, sore throat, runny nose, chest pain, nausea and vomiting, diarrhea, abdominal pain, headache, weakness, fatigue, and body ache. In addition, initial ED vital signs were recorded. We also reviewed the initial laboratory results, including complete blood count (CBC); liver function tests (LFTs); renal function; serum electrolyte levels; coagulation profile; and levels of cardiac enzymes, procalcitonin, lactic acid, lactate dehydrogenase (LDH), CRP, ferritin, and D-dimer. Finally, the outcome measures were either discharge or death due to COVID-19.

Data cleansing and preparation were performed electronically using Google Forms and Microsoft Excel. Analysis was carried out using the SPSS Statistics software version 23 (IBM, Armonk, NY). Based on our hospital reference ranges, continuous numerical variables, such as initial ED heart rate, were categorized into low, normal, and high readings. Frequencies (with percentages) and means (with standard deviations) were used for categorical and continuous variables, respectively. Binary logistic regression was performed to examine this association. A p-value of <0.05 was considered statistically significant.

## Results

A total of 656 patients were diagnosed with COVID-19 and admitted to our hospital during the study period. The mean age of the patients was 50 ± 19.4 years, and only 146 (22.2%) of the cohort were Saudi nationals. A total of 125 (19%) participants required mechanical ventilation support, and 127 (19.3%) needed ICU admission. Most patients in our population presented to the hospital in the month of May (363, 55.4%), followed by June (235, 35.8%). The majority (524, 79.9%) recovered from COVID-19 and were discharged from the hospital, while 132 (20.1%) did not recover and died. More details regarding demographic information are presented in Table 1.

**Table 1:** Demographic characteristics ICU: intensive care unit; SD: standard deviation

Characteristics		Total, n=656 (100%)	Discharged, n=524 (79.9%)	Died, n=132 (20.1%)
Age (years), mean ± SD		50.7 ± 19.4	48.7 ± 19.2	58.7 ± 18.1
Gender	Male	415 (63.3%)	319 (76.9%)	96 (23.1%)
	Female	241 (36.7%)	205 (85.1%)	36 (14.9%)
Nationality	Saudi	146 (22.2%)	136 (93.1%)	10 (6.9%)
	Non-Saudi	510 (77.8%)	388 (76.1%)	122 (23.9%)
Month of presentation	March	6 (0.9%)	5 (83.3%)	1 (16.7%)
	April	52 (7.9%)	45 (86.5%)	7 (13.5%)
	May	363 (55.4%)	295 (81.3%)	68 (18.7%)
	June	235 (35.8%)	179 (76.2%)	56 (23.8%)
Mechanical ventilation requirement	Yes	125 (19%)	32 (25.6%)	93 (74.4%)
	No	531 (81%)	492 (92.6%)	39 (7.4%)
Admission	Isolation ward	529 (80.7%)	481 (90.9%)	48 (9.1%)
	ICU	127 (19.3%)	43 (33.8%)	84 (66.2%)

A total of 274 (41.8%) participants had no prior medical history; among the rest, the most common comorbidities in descending order of prevalence were as follows: cardiovascular disease (263, 40.2%), diabetes mellitus (235, 35.9%), renal disease (51, 7.8%), cancer (23, 3.5%), asthma (23, 3.5%), and immunocompromised status (20, 3%). In addition, only 19 (2.9%) patients were smokers. Table 2 reports the medical history of the participants and compares the two groups.

**Table 2:** Past medical history COPD: chronic obstructive pulmonary disease; DM: diabetes mellitus; OHG: oral hypoglycemic

Characteristics		Total, n=656 (100%)	Discharged, n=524 (79.9%)	Died, n=132 (20.1%)
Comorbidities	No comorbidities	274 (41.8%)	248 (90.5%)	26 (9.5%)
	Cardiovascular	263 (40.2%)	184 (70%)	79 (30%)
	DM	235 (35.9%)	163 (69.4%)	72 (30.6%)
	Cancer	23 (3.5%)	13 (56.5%)	10 (43.5%)
	Asthma	23 (3.5%)	21 (91.3%)	2 (8.7%)
	Immunocompromised	20 (3%)	14 (70%)	6 (30%)
	Renal disease	51 (7.8%)	38 (74.5%)	13 (25.5%)
	Liver disease	14 (2.1%)	9 (64.3%)	5 (35.7%)
	COPD	8 (1.2%)	7 (87.5%)	1 (12.5%)
Smoker	Yes	19 (2.9%)	18 (94.7%)	1 (5.3%)
	No	637 (97.1%)	506 (79.4%)	131 (20.6%)
DM treatment	Insulin	62 (9.5%)	45 (72.6%)	17 (27.4%)
	OHG	62 (9.5%)	47 (75.8%)	15 (24.2%)
	Both	20 (3%)	12 (60%)	8 (40%)
	Not on medication	15 (2.2%)	9 (60%)	6 (40%)

The most common presenting symptom among COVID-19 patients was fever (491, 74.8%). followed by cough (420, 64%), and dyspnea (358, 54.6%). These were followed by nausea and vomiting (112, 17.1%), sore throat (97, 14.8%), and diarrhea (83, 12.6%). Abdominal pain and headache were reported at a similar frequency (66, 10.1%). Other symptoms were less frequently reported and included fatigue (60, 9.1%), chest pain (56, 8.5%), runny nose (44, 6.7%), and body ache (36, 5.5%). The least common symptom was weakness (8, 1.2%). Table 3 summarizes the presenting symptoms, frequencies, and percentages.

**Table 3:** Presenting complaints

Characteristics	Total, n=656 (100%)	Discharged, n=524 (79.9%)	Died, n=132 (20.1%)
Fever	491 (74.8%)	398 (81%)	93 (19%)
Cough	420 (64%)	345 (82.1%)	75 (17.9%)
Runny nose	44 (6.7%)	43 (97.8%)	1 (2.2%)
Sore throat	97 (14.8%)	89 (91.7%)	8 (8.3%)
Dyspnea	358 (54.6%)	273 (76.3%)	85 (23.7%)
Chest pain	56 (8.5%)	51 (91.1%)	5 (8.9%)
Nausea and vomiting	112 (17.1%)	93 (83%)	19 (17%)
Abdominal pain	66 (10.1%)	58 (87.9%)	8 (12.1%)
Diarrhea	83 (12.6%)	76 (91.6%)	7 (8.4%)
Headache	66 (10.1%)	65 (98.5%)	1 (1.5%)
Weakness	8 (1.2%)	5 (62.5%)	3 (37.5%)
Fatigue	60 (9.1%)	50 (83.3%)	10 (16.7%)
Body ache	36 (5.5%)	32 (88.9%)	4 (11.1%)

As for the vital signs measured at the initial patient contact, most of our participants had a normal temperature (357, 54.5%), normal pulse rate (319, 48.6%), and normal oxygen saturation (441, 67.2%). High systolic blood pressure and high respiratory rate were reported in 257 (39.2%) and 370 (56.4%) patients, respectively. Of note, 251 (38.3%) patients had a normal body mass index, with a mean bodyweight of 71.1 ± 18.6 kg and a mean height of 161.7 ± 17.5 cm. The initial vital signs are listed in Table 4.

**Table 4:** Initial vital signs BMI: body mass index; HTN: hypertension; SD: standard deviation

Characteristics		Total, n=656 (100%)	Discharged, n=524 (79.9%)	Died, n=132 (20.1%)
Initial temperature (°C)	Low (<36.4)	65 (9.9%)	53 (81.5%)	12 (18.5%)
	Normal (36.4–37.6)	357 (54.5%)	299 (83.7%)	58 (16.3%)
	High (>37.6)	214 (32.6%)	159 (74.3%)	55 (25.7%)
Initial pulse rate (beats/minute)	Low (<60)	4 (0.6%)	4 (100%)	0 (0%)
	Normal (60–100)	319 (48.6%)	263 (82.4%)	56 (17.5%)
	High (>100)	305 (46.5%)	237 (77.7%)	68 (22.3%)
Initial systolic blood pressure (mmHg)	Normal (100–120)	160 (24.4%)	125 (78.1%)	35 (21.9%)
	Pre-HTN (120–140)	207 (31.5%)	171 (82.6%)	36 (17.4%)
	High (>140)	257 (39.2%)	206 (80.1%)	51 (19.9%)
Initial respiratory rate (breaths/minute)	Low (<12)	2 (0.3%)	2 (100%)	0 (0%)
	Normal (12–20)	257 (39.2%)	224 (87.2%)	33 (12.8%)
	High (>20)	370 (56.4%)	281 (76%)	89 (24%)
Initial oxygen saturation (%)	Low (<95)	192 (29.3%)	116 (60.4%)	76 (39.6%)
	Normal (>95)	441 (67.2%)	393 (89.1%)	48 (10.9%)
BMI (Kg/m^2^)	Underweight (<18.5)	26 (4%)	19 (73%)	7 (27%)
	Normal (18.5–24.9)	251 (38.3%)	200 (79.7%)	51 (20.3%)
	Overweight (25–29.9)	199 (30.3%)	157 (78.9%)	42 (21.1%)
	Obese (>29.9)	151 (23%)	123 (81.5%)	28 (18.5%)
Weight (Kg), mean ± SD		71.1 ± 18.6	70.5 ± 17.8	73.1 ± 21.6
Height (cm), mean ± SD		161.7 ± 17.5	162 ± 15.5	160.3 ± 23.5

Laboratory investigations conducted initially upon patient admission showed that most of the participants (405, 63.6%) had a normal white blood cell count, normal hemoglobin levels (405, 61.7%), normal platelet count (524, 79.9%), normal alkaline phosphatase levels (528, 80.5%), normal bilirubin levels (588, 89.6%), normal blood urea nitrogen levels (433, 66%), normal creatinine levels (477, 72.7%), normal troponin levels (408, 62.2%), normal international normalized ratio (474, 72.2%), and normal procalcitonin levels (270, 41.1%). On the other hand, 519 (79.1%) had low albumin levels, 517 (78.8%) had high CRP levels, and 304 (46.3%) had high ferritin levels. Table 5 provides more details about the participants’ laboratory characteristics.

**Table 5:** Initial laboratory characteristics ALT: alanine transaminase; ALP: alkaline phosphatase; AST: aspartate aminotransferase; CRP: C-reactive protein; GGT: gamma-glutamyl transferase; INR: international normalized ratio; LDH: lactate dehydrogenase; PTT: partial thromboplastin time; PT: prothrombin time; WBC: white blood cell

Characteristics		Total, n=656 (100%)	Discharged, n=524 (79.9%)	Died, n=132 (20.1%)
WBC count (K/uL)	Low (<4.5)	135 (20.6%)	121 (89.6%)	14 (10.4%)
	Normal (4.5–11.5)	417 (63.6%)	347 (83.2%)	70 (16.8%)
	High (>11.5)	99 (15.1%)	51 (51.5%)	48 (48.5%)
Neutrophil count (K/uL)	Low (<2)	76 (11.6%)	71 (93.4%)	5 (6.6%)
	Normal (2–7.5)	447 (68.1%)	375 (83.9%)	72 (16.1%)
	High (>7.5)	121 (18.4%)	66 (54.5%)	55 (45.5%)
Lymphocyte count (K/uL)	Low (<1.5)	388 (59.1%)	303 (78.1%)	85 (21.9%)
	Normal (1.5–4)	241 (36.7%)	200 (83%)	41 (17%)
	High (>4)	12 (1.8%)	6 (50%)	6 (50%)
Monocyte count (K/uL)	Low (<0.4)	212 (32.3%)	166 (78.3%)	46 (21.7%)
	Normal (0.4–1)	364 (55.5%)	296 (81.3%)	68 (18.7%)
	High (>1)	55 (8.4%)	39 (70.9%)	16 (29.1%)
Eosinophil count (K/uL)	Low (<0.04)	355 (54.1%)	289 (81.4%)	66 (18.6%)
	normal (0.04–0.4)	272 (41.5%)	213 (78.3%)	59 (21.7%)
	High (>0.4)	15 (2.3%)	9 (60%)	6 (40%)
Platelet count (K/uL)	Low (<150)	101 (15.4%)	66 (65.3%)	35 (34.7%)
	Normal (150–450)	524 (79.9%)	432 (82.4%)	92 (17.6%)
	High (>450)	26 (4%)	21 (80.8%)	5 (19.2%)
Hemoglobin (g/dL)	Low (<12)	225 (34.3%)	152 (67.6%)	73 (32.4%)
	Normal (12–16)	405 (61.7%)	349 (86.2%)	56 (13.8%)
	High (>16)	20 (3%)	17 (85%)	3 (15%)
ALT (U/L)	Normal (0–50)	413 (63%)	333 (80.6%)	80 (19.4%)
	High (>50)	209 (31.8%)	158 (75.6%)	51 (24.4%)
AST (U/L)	normal (10–40)	345 (52.6%)	307 (89%)	38 (11%)
	High (>40)	261 (39.8%)	169 (64.8%)	92 (35.2%)
ALP (U/L)	Normal (40–150)	528 (80.5%)	435 (52.7%)	93 (47.3%)
	High (>150)	78 (11.9%)	41 (52.6%)	37 (47.4%)
GGT (U/L)	Normal (0–55)	369 (56.2%)	308 (83.5%)	61 (16.5%)
	High (>55)	254 (38.7%)	184 (72.4%)	70 (27.6%)
Bilirubin (μmol/L)	Normal (3–22)	588 (89.6%)	475 (80.8%)	113 (19.2%)
	High (>22)	34 (5.2%)	16 (47.1%)	18 (52.9%)
Albumin (g/L)	Low (<40.2)	519 (79.1%)	392 (75.5%)	127 (24.5%)
	Normal (40.2–47.6)	81 (12.3%)	77 (95.1%)	4 (4.9%)
	High (>47.6)	10 (1.5%)	10 (100%)	0 (0%)
Urea (mmol/L)	Normal (2.5–6.4)	433 (66%)	396 (91.4%)	37 (8.6%)
	High (>6.4)	214 (32.6%)	119 (55.6%)	95 (44.4%)
Creatinine (umol/L)	Normal (53–115)	477 (72.7%)	420 (88%)	57 (12%)
	High (>115)	170 (25.9%)	95 (55.9%)	75 (44.1%)
Sodium (mmol/L)	Low (<136)	259 (39.5%)	196 (75.7%)	63 (24.3%)
	Normal (136–145)	367 (55.9%)	310 (84.5%)	57 (15.5%)
	High (>145)	18 (2.7%)	6 (33.3%)	12 (66.7%)
Potassium (mmol/L)	Low (<3.5)	144 (21.9%)	118 (81.9%)	26 (18.1%)
	Normal (3.5–5.1)	473 (72.1%)	379 (80.1%)	94 (10.9%)
	High (>5.1)	27 (4.1%)	15 (55.6%)	12 (44.4%)
Troponin (ug/L)	Normal (0.02–0.04)	408 (62.2%)	350 (85.8%)	58 (14.2%)
	High (>0.04)	111 (16.9%)	45 (40.5%)	66 (59.5%)
PT (seconds)	Normal (9.4–12.5)	326 (49.7%)	272 (83.4%)	54 (16.6%)
	High (>12.5)	226 (34.4%)	149 (65.9%)	77 (34.1%)
PTT (seconds)	Normal (25.1–36.5)	434 (66.1%)	345 (79.5%)	89 (20.5%)
	High (>36.5)	104 (15.8%)	64 (61.5%)	40 (38.5%)
INR	Normal (0.85–1.3)	474 (72.2%)	388 (81.8%)	86 (18.6%)
	High (>1.3)	79 (12%)	35 (44.3%)	44 (55.7%)
Lactic acid (mmol/L)	Normal (0.4–2)	174 (26.5%)	116 (66.7%)	58 (33.3%)
	High (>2)	87 (13.2%)	32 (36.8%)	55 (63.2%)
D-dimer (mg/L)	Normal (≤0.5)	189 (28.8%)	177 (93.6%)	12 (6.4%)
	High (>0.5)	87 (13.3%)	32 (36.8%)	55 (63.2%)
LDH (U/L)	Normal (100–240)	125 (19%)	116 (92.8%)	9 (7.2%)
	High (>240)	310 (47.2%)	203 (65.5%)	107 (34.5%)
CRP (mg/L)	Normal (≤3)	3 (0.4%)	3 (100%)	0 (0%)
	High (>3)	517 (78.8%)	396 (76.6%)	121 (23.4%)
Ferritin (ng/mL)	Normal (20–250)	191 (29.1%)	183 (95.8%)	8 (4.2%)
	High (>250)	304 (46.3%)	192 (63.1%)	112 (36.9%)
Procalcitonin (ng/mL)	Normal (≤0.15)	270 (41.1%)	258 (95.5%)	12 (4.5%)
	High (>0.15)	196 (29.9%)	105 (53.6%)	91 (46.4%)
Fibrinogen (mg/dL)	Normal (200–400)	81 (12.3%)	60 (74%)	21 (26%)
	High (>400)	128 (19.5%)	61 (47.6%)	67 (52.4%)
Random glucose (mmol/L)	Low (<4.4)	11 (1.7%)	6 (54.5%)	5 (45.5%)
	Normal (4.4–7.8)	182 (27.7%)	131 (72%)	51 (28%)
	High (>7.8)	168 (25.6%)	105 (62.5%)	63 (37.5%)
HbA1C (%)	Normal (≤6.5)	62 (9.4%)	53 (85.5%)	9 (14.5%)
	High (>6.5)	122 (18.6%)	89 (72.9%)	33 (27.1%)

Some factors were found to be significantly associated with in-hospital deaths among admitted COVID-19 patients. Patients more likely to die were those of advanced age (OR: 1.018; 95% CI: 1.006-1.031; p=0.005); male gender (OR: 1.919; 95% CI: 1.179-3.123; p=0.009); non-Saudi nationality (OR: 3.937; 95% CI: 1.866-8.301; p<0.001); those with low platelet (OR: 1.005; 95% CI: 1.001-1.010; p=0.015), low hemoglobin levels (OR: 1.301; 95% CI: 1.085-1.559; p=0.004), low albumin levels (OR: 1.115; 95% CI: 1.037-1.200; p=0.003), high aspartate aminotransaminase levels (OR: 1.006; 95% CI: 1.000-1.012; p=0.039), high alkaline phosphatase levels (OR: 1.011; 95% CI: 1.004-1.017; p=0.002), high blood urea nitrogen levels (OR: 1.196; 95% CI: 1.109-1.290; p<0.001), high partial thrombin time (OR: 1.029; 95% CI: 1.002-1.060; p=0.035), and high LDH levels (OR: 1.006; 95% CI: 1.003-1.008; p<0.001). Additionally, patients who required mechanical ventilation support were more likely to die than non-mechanically ventilated patients (OR: 18.924, 95% CI: 8.235-43.48; p<0.001). On the other hand, patients who presented with chest pain (OR: 0.249; 95% CI: 0.088-0.705; p=0.009) or headache (OR: 0.058; 95% CI: 0.007-0.462; p=0.007) or had low eosinophil counts (OR: 0.090; 95% CI: 0.011-0.719; p=0.023), high gamma-glutamyl transferase levels (OR: 0.995; 95% CI: 0.990-1.000; p=0.034), or high creatinine levels (OR: 0.994; 95% CI: 0.991-0.998; p=0.001) were significantly less likely to die. The results of the binary logistic regression test are summarized in Table 6.

**Table 6:** Binary logistic regression test to study variables associated with death ALT: alanine transaminase; ALP: alkaline phosphatase; AST: aspartate aminotransferase; BMI: body mass index; COPD: chronic obstructive pulmonary disease; CRP: C-reactive protein; DM: diabetes mellitus; GGT: gamma-glutamyl transferase; INR: international normalized ratio; LDH: lactate dehydrogenase; PTT: partial thromboplastin time; PT: prothrombin time; WBC: white blood cell

Characteristics	P-value	Odds ratio	95% confidence interval
Advanced age	0.005	1.018	1.006–1.031
Male gender	0.009	1.919	1.179–3.123
Non-Saudi nationality	0.000	3.937	1.866–8.301
No comorbidities	0.165	0.576	0.265–1.254
Known cardiovascular disease	0.623	1.177	0.615–2.252
Known DM	0.071	1.934	0.944–3.959
Known cancer	0.062	3.314	0.943–11.64
Known asthma	0.340	0.429	0.075–2.445
Known immunocompromised state	0.986	0.989	0.287–3.409
Known renal disease	0.470	0.754	0.350–1.623
Known liver disease	0.816	1.179	0.294–4.724
Known COPD	0.416	0.399	0.044–3.638
Known smoker	0.120	0.177	0.020–1.568
Required mechanical ventilation	0.000	18.924	8.235–43.48
High BMI	0.612	0.981	0.911–1.055
Having fever	0.961	0.987	0.589–1.655
Having cough	0.257	0.663	0.407–1.080
Having runny nose	0.162	0.228	0.029–1.807
Having sore throat	0.223	0.590	0.253–1.377
Having dyspnea	0.382	1.242	0.764–2.018
Having chest pain	0.009	0.249	0.088–0.705
Having nausea and vomiting	0.803	1.090	0.552–2.155
Having abdominal pain	0.979	1.013	0.388–2.644
Having diarrhea	0.419	0.684	0.272–1.719
Having headache	0.007	0.058	0.007–0.462
Having weakness	0.818	1.221	0.223–6.699
Having fatigue	0.745	0.872	0.383–1.985
Having body ache	0.799	1.177	0.335–4.133
High temperature	0.711	0.990	0.938–1.043
High pulse rate	0.489	0.993	0.972–1.031
Low systolic blood pressure	0.370	0.993	0.978–1.008
High respiratory rate	0.653	0.984	0.919–1.053
Low initial oxygen saturation	0.147	1.038	0.987–1.093
Low WBC count	0.138	0.924	0.832–1.026
Low neutrophil count	0.504	0.955	0.834–1.094
Low lymphocyte count	0.631	0.989	0.944–1.036
Low monocyte count	0.136	2.245	0.775–6.503
Low eosinophil count	0.023	0.090	0.011–0.719
Low platelet count	0.015	1.005	1.001–1.010
Low hemoglobin	0.004	1.301	1.085–1.559
High ALT	0.124	0.989	0.974–1.003
High AST	0.039	1.006	1.000–1.012
High ALP	0.002	1.011	1.004–1.017
High GGT	0.034	0.995	0.990–1.000
High bilirubin	0.313	1.005	0.995–1.015
Low albumin	0.003	1.115	1.037–1.200
High urea	0.000	1.196	1.109–1.290
High creatinine	0.001	0.994	0.991–0.998
High sodium	0.742	0.992	0.947–1.039
High potassium	0.884	0.993	0.898–1.096
High troponin	0.646	1.035	0.892–1.202
High PT	0.173	1.035	0.985–1.087
High PTT	0.035	1.029	1.002–1.060
High INR	0.122	1.036	0.990–1.084
High lactic acid	0.421	1.008	1.095–1.027
High D-dimer	0.318	1.021	0.980–1.065
High LDH	0.000	1.006	1.003–1.008
High CRP	0.563	1.002	0.996–1.007
High ferritin	0.070	1.000	1.000–1.000
High procalcitonin	0.155	1.074	0.972–1.186
Low random glucose	0.500	1.039	0.930–1.160

## Discussion

The aim of this study was to predict the in-hospital outcomes among COVID-19 patients (recovery and discharge or death) depending on the patients’ initial characteristics. Based on our findings, the in-hospital outcomes of admitted COVID-19 patients vary depending on many factors, such as a history of chronic diseases, initial vital signs, laboratory investigations, and other demographic factors. The majority of the admitted patients enrolled in this study recovered and were discharged from the hospital, and only 20% of the patients died due to multiple risk factors.

The mean age of the admitted patients was 50 ± 19.4 years, and a majority of the patients who recovered and were discharged were younger (48.7 ± 19.2) than those who died (58.7 ± 18.1). In fact, older patients are more vulnerable to severe infection than younger patients, possibly due to many risk factors such as comorbidities, polypharmacy, and suppressed immunity, all of which predispose older patients to severe conditions and higher mortality rates compared to younger age groups [9,10]. Moreover, according to an important observation about COVID-19 patients by the WHO Regional Director for the European region, older patients are at a greater risk of developing severe infections, and the estimated death rate in older people over the age of 60 years is 95% [11]. The majority of our patients were male, which was in line with some previous studies that reported that males were more susceptible to COVID-19 infection than females [12,13].

In this study, only a minority of our patients required mechanical ventilation (19%) and ICU admission (19.3%). However, the mortality rate was higher among those patients who required mechanical ventilation (74%) than among non-mechanically ventilated patients. Moreover, patients admitted to the ICU had a higher mortality rate (66%) than those admitted to the isolation ward. More severe cases typically require respiratory support and ICU care; thus, the higher death rate among those patients is more likely to be associated with the severity of the disease. A retrospective study that involved the collection of data from nine tertiary hospitals in the Hubei province, China, reported that the higher death rate in hospitals might be associated with the fact that most of the admitted cases had more severe disease, presented at the late stage of the disease, and had more complications than home-quarantined people [12,14].

As mentioned previously, most admitted patients have multiple risk factors and are more predisposed to the complications of COVID-19. The majority of the admitted patients had existing comorbidities (58.2%). The most common comorbidity was cardiovascular disease, which was found to increase the risk of death and complications among COVID-19 patients [15]. The second most common comorbidity was diabetes mellitus, which was also associated with a higher mortality rate among infected patients, and it increased the risk of mortality by two-fold compared to non-diabetic patients [16]. Other common comorbidities included renal disease, cancer, asthma, and immunocompromised status. Patients with comorbidities are at a greater risk of severe complications and higher mortality rates than those with no comorbidities [17].

The most common presenting symptoms in our patients were fever, cough, dyspnea, nausea and vomiting, sore throat, and diarrhea. Generally, upper respiratory symptoms are more common and predominate over gastrointestinal symptoms, as reported in other studies [18]. In line with the findings of other studies, fever was the most common presenting symptom in our study too. However, it is important to note that not all patients presented with fever on admission [19,13]. The presenting symptoms could vary from patient to patient, and there is no clear relationship between specific presenting symptoms and the severity of the COVID-19 infection or the general clinical outcomes [20].

On the other hand, the majority of our patients had normal vital signs upon arrival at the ED, with the exception of an elevated respiratory rate and systolic blood pressure. This is similar to many other studies that have reported respiratory rate impairment in COVID-19 patients; it is expected given the respiratory nature of the disease [21,22]. That said, impairments range from milder forms usually encountered during emergency presentations to more severe forms like those seen in patients in the ICU and intubated patients [23].

Similar to vital signs, most of our patients had normal laboratory findings at the time of presentation; CBC, kidney function, electrolytes, LFTs except low albumin, coagulation profile, and troponin-I were all normal. Several studies have endorsed this finding of normal labs overall, as reported by Tan et al. [3], Shi et al. [22], Yue et al. [7], and many others. However, this was contrasted by the findings of Luo et al., who showed that patients had below-normal leukocyte and lymphocyte counts [24]. In addition, He et al. found that patients had derangements in coagulation profile studies at the time of presentation [25]. However, not all laboratory findings were normal in our study; elevated levels of CRP and ferritin were noted in the initial measurements taken in the ED for the majority of our patients, which could be explained by the fact that the levels of these inflammatory markers could be influenced by disease activity early in the course of the disease, as mentioned by a few other papers [5,21,26].

This variation in laboratory results between different studies have led to a widely varied perception of the importance of laboratory features; while some studies have found that laboratory features were not the main predictors of COVID-19 outcomes [3], others have reported that some laboratory features were the major and best predictors for COVID-19 outcomes [26]. Still, it should be noted that for our patients, many laboratory features were strongly associated with a worse prognosis of COVID-19, such as low hemoglobin levels, low platelet counts, low albumin levels, elevated liver enzyme levels, prolonged prothrombin time, and elevated LDH levels. Although some of these laboratory features might not be directly associated with the COVID-19 disease mechanism, it does not change the fact that they could be used as predictors of overall patient outcomes. Finally, we think that the best way of settling the above-mentioned conflict between papers is to take all the prognostic factors collectively into consideration for a single patient, while not ignoring the clinical features and not overly depending on the laboratory features.

Limitations and recommendations

Several limitations were encountered in our research, e.g., the non-availability of some laboratory features for some patients as not all laboratory exams were performed for all patients; however, we tried to increase our sample size to overcome any effect of such missing laboratory tests on the statistical value of our variables. It should also be noted that this was a single-center study carried out over a period of four months, and we recommend further studies involving wider areas and covering many centers and preferably many patient groups to achieve optimal statistical results.

## Conclusions

Based on our findings among participants with COVID-19, older male patients with low platelet counts, low hemoglobin levels, low albumin levels, high alkaline phosphatase levels, high blood urea nitrogen levels, high LDH levels, tachypnea, and those requiring mechanical ventilation were more likely to have a complicated course of illness and die eventually. Therefore, an understanding of the demographic, clinical, and laboratory features is essential to determine the overall in-hospital outcomes of admitted COVID-19 patients, and this could help clinicians to identify more severe cases and provide the best possible interventions to ameliorate in-hospital outcomes.
